# Inferring Gene-Phenotype Associations via Global Protein Complex Network Propagation

**DOI:** 10.1371/journal.pone.0021502

**Published:** 2011-07-25

**Authors:** Peng Yang, Xiaoli Li, Min Wu, Chee-Keong Kwoh, See-Kiong Ng

**Affiliations:** 1 Bioinformatics Research Centre, School of Computer Engineering, Nanyang Technological University, Singapore, Singapore; 2 Department of Data Mining, Institute for Infocomm Research, Agency for Science, Technology and Research, Singapore, Singapore; Aarhus University, Denmark

## Abstract

**Background:**

Phenotypically similar diseases have been found to be caused by functionally related genes, suggesting a modular organization of the genetic landscape of human diseases that mirrors the modularity observed in biological interaction networks. Protein complexes, as molecular machines that integrate multiple gene products to perform biological functions, express the underlying modular organization of protein-protein interaction networks. As such, protein complexes can be useful for interrogating the networks of phenome and interactome to elucidate gene-phenotype associations of diseases.

**Methodology/Principal Findings:**

We proposed a technique called RWPCN (Random Walker on Protein Complex Network) for predicting and prioritizing disease genes. The basis of RWPCN is a protein complex network constructed using existing human protein complexes and protein interaction network. To prioritize candidate disease genes for the query disease phenotypes, we compute the associations between the protein complexes and the query phenotypes in their respective protein complex and phenotype networks. We tested RWPCN on predicting gene-phenotype associations using leave-one-out cross-validation; our method was observed to outperform existing approaches. We also applied RWPCN to predict novel disease genes for two representative diseases, namely, Breast Cancer and Diabetes.

**Conclusions/Significance:**

Guilt-by-association prediction and prioritization of disease genes can be enhanced by fully exploiting the underlying modular organizations of both the disease phenome and the protein interactome. Our RWPCN uses a novel protein complex network as a basis for interrogating the human phenome-interactome network. As the protein complex network can capture the underlying modularity in the biological interaction networks better than simple protein interaction networks, RWPCN was found to be able to detect and prioritize disease genes better than traditional approaches that used only protein-phenotype associations.

## Introduction

Uncovering the associations between the genetic diseases and their causative genes is a fundamental objective of human genetics [1]. However, despite the recent genomic revolution, it still remains a daunting challenge because of the pleiotropy of genes, the limited number of phenotype-gene associations, the genetic heterogeneity of diseases, as well as other complications [2], [3].

In recent years, there has been an increase in the number of genes confirmed as causative genes to diseases [4]. Such information can be exploited by computational methods to predict or prioritize new disease-gene associations. A common approach is to measure the similarities between the candidate genes and the known disease causative genes based on biological evidences of these genes such as protein sequence information [5], gene expression profiles [6], and even literature descriptions [7]. Candidate genes that share high similarities with the known disease causative genes can then be ranked as the putative disease genes to be validated by biologists or clinicians. However, these approaches are limited by the quality and completeness of the biological evidences. They are also not very useful for inferring causative genes for new diseases, for it will depend not only on the accuracy of the biological similarities of the genes being compared, but also on the ability to categorize similar diseases correctly. Given that different diseases encompass different but sometime overlapping collections of clinical phenotypes, a more viable approach would be to link or prioritize the candidate genes based on the clinical manifestations of the diseases, that is, to identify gene-phenotype associations instead of gene-disease associations directly.

Recent studies have revealed that similar phenotypes are often caused by functionally related genes [8], [9], and genes associated with similar disorders have been shown to demonstrate higher probability of physical interactions between their gene products [10], [11]. This suggests a guilt-by-association prediction and prioritization of disease genes by interrogating the networks of phenome and interactome for correlations that elucidate gene-phenotype associations of diseases. A graphical map of the phenome can be constructed by considering each phenotype as a node, and then linking the highly similar phenotypes (e.g. based on the similarity in their corresponding OMIM records or the domain knowledge form clinicians). On the other hand, a graphical map of the interactome can be constructed more directly by considering each individual protein as a node, and the existence of an experimentally detected protein-protein interaction (PPI) as a link between the two corresponding nodes. The recent advent in high-throughput methods for detecting PPIs en masse (e.g. yeast-two-hybrid, co-immunoprecipitation-coupled mass spectroscopy) has enabled the construction of PPI networks on a genomic scale. With the two networks, we can then infer gene-phenotype associations by computing the closeness between the candidate genes and known disease genes based on network topological properties [3], [12]–[14]. For example, Wu *et al.* [15] built a regression model measuring the correlation between phenotype similarities and gene closeness in the PPI network for prioritizing candidate disease genes based on the correlation scores. However, Wu's method is limited by the consideration of only small localized regions in both the protein interaction network and phenotype network. To address this issue, Vanunu and Sharan [16] designed a global network-based method by formulating constraints on the genes' score function that related to its smoothness over the network. Most recently, Li and Patra [3] proposed a new method to prioritize disease genes by extending the random walk with restart algorithm on a heterogeneous network constructed by connecting the gene network (i.e. protein interaction network) and the phenotype network using known phenotype-gene relationships.

The observation that phenotypically similar diseases are often caused by functionally related genes also suggests a modular organization of the genetic landscape of human diseases. Many specific examples have shown that individual genes that cause a given phenotype tend to be linked at the biological levels as components of a multi-protein complex [10], [11]. In other words, the causative genes for the same or phenotypically similar diseases are likely to reside in the same biological module. Protein complexes, as molecular machines that integrate multiple gene products to perform biological functions, are direct manifestations of biological modules. They are also detected as tightly linked substructures in PPI networks [17], reflecting the modularity of biological networks graphically. As such, protein complexes can be a useful basis for interrogating the networks of phenome and interactome to elucidate gene-phenotype associations of diseases.

Both the Vanunu and Li methods mentioned above did not make use of protein complexes to aid in their inference of gene-phenotype associations. In an earlier work, Lage *et al.* [18] made use of protein complexes for prioritization of disease genes via phenotypic weighting of protein complexes linked to human diseases. However, they did not use actual protein complexes but simply assembled neighboring proteins as complexes (consist of a protein and all their direct interaction partners). They also ignored the biological relationships between the protein complexes. For example, it has been reported that if two protein complexes share a number of common proteins or have densely physical interactions between them, the mutations of genes in one protein complex could lead to same or similar phenotypes of the other protein complex [11]. As such, incorporating quality-controlled protein complexes and accounting for their relationships are both essential for accurate disease gene prediction. In this work, we therefore propose to construct a novel protein complex network, where nodes are individual complexes and the interactions between two complexes are measured by the connection strengths between them, as a basis for interrogating the phenome-interactome networks for disease gene prioritization. We devise a novel globally network-based technique called RWPCN (Random Walker on Protein Complex Network) for elucidating novel gene-phenotype relationships on such a network.

Our proposed method is different from the existing methods as our network propagation algorithm is operated at the complex-level instead of the protein level. We used reliable human protein complexes from the Comprehensive Resource of Mammalian protein complexes (CORUM) [19] since the protein complexes were curated from the biological literatures. To our best knowledge, this is the first attempt to capture and exploit the biological modularity of the protein complexes and their relationships in an explicit way. Our experimental results showed that such an effort was indeed worth the while, for our proposed algorithm was able to discover gene-disease associations more effectively as compared with existing state-of-the-art methods.

## Materials and Methods

In this section, we will first describe the experimental data we have used. Then, we will introduce the overall network structure for our RWPCN algorithm, including the phenotype network, protein complex network, protein interaction network, as well as gene-phenotype associations. Finally, we describe the construction of the phenotype network and protein complex network. With these, we then present our RWPCN algorithm for prioritizing disease-related genes.

### Data Set

#### Protein interaction data

Human PPI data were downloaded from the Human Protein Reference database (HPRD) [20] database which has 34364 interactions among 8919 human proteins/genes. We filtered out the proteins with only self-interactions, resulting in 8756 human proteins.

#### Protein complex data

Human protein complexes data were downloaded from the Comprehensive Resource of Mammalian protein complexes (CORUM) database [19]. The CORUM database is a collection of experimentally verified mammalian protein complexes and these protein complexes are manually extracted from literature. The records of CORUM protein complexes are generated by different kinds of experiments, such as *coimmunoprecipitation*, *cosedimentation*, and *ion exchange chromatography*. We only considered those human protein complexes that include at least one gene in HPRD human protein interaction data. This resulted in 379 human protein complexes with an average size (the average number of proteins) of 3.83. This set of protein complexes contains a total of 918 human genes, covering 10.5% of human genes in our PPI network. Note that we have also filtered out giant complexes if they covered a number of smaller complexes.

Recall that we also view those proteins which are not included in any CORUM protein complexes as individual protein complexes. There were 7838 of these, and 3964 of these individual protein complexes directly interact with CORUM complexes in our resulting protein complex network.

#### Gene-phenotypeassociations

Gene-phenotype associations are assembled from the OMIM database [4], using BIOMART [21]. In our experiments, we exploited an old version of gene-phenotype association data used in previous studies [3], [15] to facilitate comparisons. 1428 known gene-phenotype associations were extracted, spanning 1126 disease phenotypes and 937 causative genes. In addition, we also collected a new version of gene-phenotype relationships from BIOMART [22], which contained 1614 links, with 1266 disease phenotypes and 1034 causative genes.

### Overall Network Structure in RWPCN

**Figure 1:**
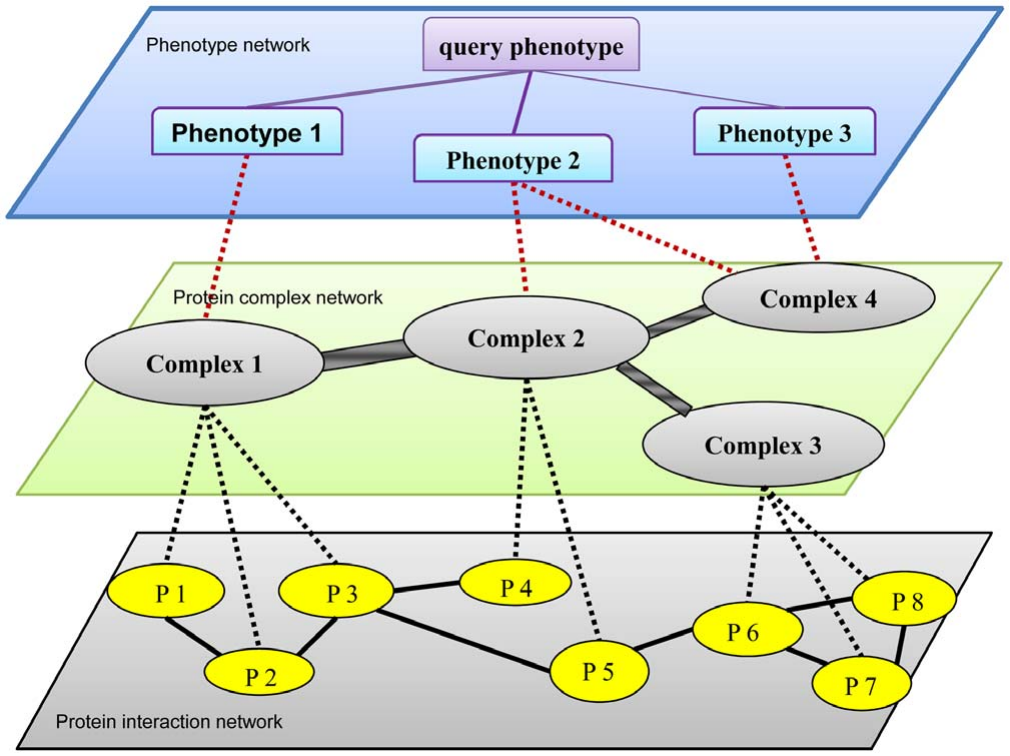
Illustration of the overall network structure in RWPCN. Top level phenotype network connects the phenotypes and query phenotype if their phenotypic similarity scores are bigger enough. The similarity scores are used to weight the links (the links are marked with purple lines, where the thicker lines denote higher phenotypic similarities). The middle level of the network is protein complex network where phenotype-related protein complexes are connected. The links between two protein complexes marked with gray lines, with the thicker lines indicate the strong linkage strength. On the other hand, the links between the phenotypes and complexes indicate the known gene-phenotype associations, denoted by dashed blue lines. The bottom level of the network is the protein interaction network where each protein complex in the middle level links with all its component proteins (yellow nodes).

Figure 1 depicts the overall network structure used in RWPCN. It consists of three levels of networks, namely, the phenotype network (top), protein complex network (middle), and the protein interaction network (bottom). In the phenotype network at the top level, we connect phenotypes if their similarity scores are bigger than a pre-defined threshold. The similarity scores are also used to weight the links. In the Figure, the links are marked with purple lines, where the thicker lines denote higher phenotypic similarities.

The protein complex network in the middle layer is where phenotypically-related protein complexes are connected. Within the protein complex networks, the links are marked with gray lines, with the thicker lines indicating stronger linkage strengths between the two corresponding protein complexes. We will describe how to compute the protein complexes' linkage strengths later. The links between the phenotypes and complexes capture the known gene-phenotype associations, denoted by dashed red lines.

At the bottom level is the PPI network. Two proteins are connected if they were reported to be interacting to each other. Across the networks, each protein complex in the middle level links with all its component proteins (yellow nodes) in the PPI network.

Given a query disease phenotype (a query node in the top level), our objective is to predict disease genes for this phenotype in the bottom level PPI network, guided by the protein complex relationships in the middle level. Our proposed RWPCN algorithm will traverse between the three networks and exploit the structural relationships accordingly.

### Constructing Phenotype Network

Biologists already have a detailed knowledge of the phenotypes that are associated with each other. These phenotype associations have been used to prioritize candidate disease genes as well as to discover functional relations between genes and proteins [23].

As in the method by van Driel [23], we construct the phenotype network by applying a text-mining approach to evaluate the similarity among OMIM phenotypes using Medical Subject Headings (*MeSH*) controlled vocabulary as standardized phenotypic feature terms. A phenotype *pt*∈*PT* (*PT* is the set of all the phenotypes) is represented as a feature vector *pt* = (*x*_1_, *x*_2_,…, *x_l_*) where each dimension represents a vocabulary of Medical Subject Headings. A dimension *i* (1< = *i*< = *l*) in the feature vector *pt* represents a *MeSH* concept which provides a standardized way to retrieve information to refer to the concept. Each feature value *x_i_* is the weight of *i* ^th^ *MeSH* concept, which is determined by the concept relevance and document frequency in [23].

Given two *pt_i_* = (*x_i_*_1_, *x_i_*_2_,…, *x_il_*), *pt_j_* = (*y_j_*_1_, *y_j_*_2_,…, *y_jl_*), we measure the phenotypic similarity between two vectors by the cosine similarity between the normalized vectors, i.e.,
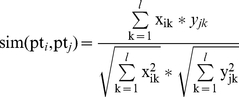
(1)As recommended in [23], similarity values in the range [0, 0.3] are believed to be uninformative and noisy while those in [0.6, 1] are considered to be reliable [23]. Therefore, we re-compute the phenotypic similarity between *pt_i_* and *pt_j_* using a logical function 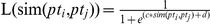 used in [24]. We used the default values recommended in [24] for the parameters *c* and *d*, namely *c = *-15 and *d = *log(9999) respectively.

We construct our phenotype network using *k-*NN model (*k*-Nearest Neighbor). That is, for each phenotype *pt_i_*, we compute its top *k* most similar phenotypic neighbors (i.e. having the *k* highest phenotypic similarities with *pt_i_*) to link to it. We experimentally test the effects of different values of *k* on the performance of our proposed algorithm, and we set *k* = 10 as the default value.

### Constructing Protein Complex Network

A PPI network (in the bottom level) is an undirected graph *G_PPI_* = (*V_PPI_*, *E_PPI_*), where *V_PPI_* is the set of nodes (proteins) and *E* = {(*u*,*v*)| *u*,*v*∈*V_PPI_*} is the set of edges (protein interactions). To construct protein complex network in the middle level, we need to collect known protein complex data or use some computational methods to predict protein complexes. In this paper, we use the known protein complex database Comprehensive Resource of Mammalian protein complexes (CORUM) [19], which is a collection of high quality experimentally verified mammalian protein complexes and has higher quality than those predicted by computational methods. However, the CORUM complex database is still far from complete and it is built from 2400 different genes, covering 12% of protein-coding genes in human [19]. As such, our protein complex set *COM* consists of a set of multi-protein complexes from CORUM (set *C_M_*) as well as a set of *individual complexes* (set *C* ***_I_***) — namely those individual proteins that are not involved in any of the current CORUM complexes. As such, we have the following:
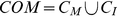
(2)

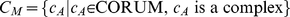
(3)

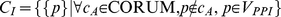
(4)


Given the protein complex set *COM*, we define the protein complex network as a directed super graph *G_COM_* = (*V_COM_*, *E_COM_*), where the super node set *V_COM_* = *COM* denotes a set of protein complexes and *E_COM_* = {(*c_A_*,*c_B_*)| *c_A_*,*c_B_*∈*V_COM_*} represents the set of links between protein complexes. Note that a link (*c_A_*,*c_B_*)∈*E_COM_* can be categorized into one of three types depending on the nature of complexes *c_A_* and *c_B_*, namely, *E_C_*_2*C*_ (*C2C* links between two multi-protein complexes), *E_I_*_2*I*_ (*I*2*I* links between two individual complexes), and *E_I_*_2*C*_ (*I*2*C* links between an individual complex and a multi-protein complex). Next, we describe how to assign weight for these three types of links.

Note that each complex *c_A_* ∈*C_M_* is a super node that can be represented as a graph *c_A_* = (*V_cA_*, *E_cA_*) where the set *V_cA_* represents all the proteins in the complex *c_A_*, and the set *E_cA_* represents the protein-protein interactions among the proteins in *V_cA_*. Given two complexes *c_A_* = (*V_cA_*, *E_cA_*) and *c_B_* = (*V_cB_*, *E_cB_*), *c_A_*,*c_B_*∈*C_M_* , a *C2C* link *E_C2C_*(*c_A_*,*c_B_*) between *c_A_* and *c_B_* can be quantified as follows:
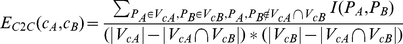
(5)where
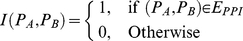
(6)


Basically, Equation (5) evaluates how closely the protein members from different complexes interact with each other overall. It is the proportion number of interaction between the complexes among the number of all possible interactions. If there are a lot of physical interactions between the members from two complexes (non-overlapping proteins), then the two complexes are likely to be highly related as mutations of proteins in one of protein complexes could correspondingly disrupt the other complexes' functions, thereby producing similar disease phenotypes. Note that according to equation (5), it is easy to know that *E_C_*_2*C*_ (*c_A_*,*c_B_*) = *E_C_*_2*C*_ (*c_B_*, *c_A_*).

In the case that we have one multi-protein complex *c_A_*∈*C_M_* and one *individual* protein complex *I_A_* ∈*C_I_*, then the C2I link *E_C_*_2*I*_ (*c_A_*, *I_A_*) and the I2C link *E_I2C_* (*I_A_*, *c_A_*) can be defined as follows:
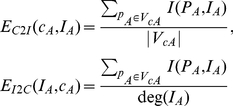
(7)


Finally, given two *individual* protein complexes *I_A_* and *I_B_*, (*I_A_*, *I_B_* ∈*C_I_*), then the I2I link *E_I_*_2*I*_ (*I_A_*, *I_B_*) and the I2I link *E_I2I_* (*I_B_*, *I_A_*) are computed as follows:
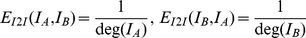
(8)where deg(*I_A_*) is the number of neighbors of vertex *I_A_*.

### Random walk with restart on the protein complexes network (RWPCN)

We are now ready to present our proposed algorithm. Given a query phenotype *pt_i_*, we aim to prioritize candidate disease genes based on known disease genes which are associated with *pt_i_*'s similar phenotypic neighbors in the phenotype network.

#### Step 1. Initialization of seed genes and complexes

Let *N*(*pt_i_*) represents the *k*-NN phenotype neighbor set of the query phenotype *pt_i_* where each *pt_j_*∈*N*(*pt_i_*) is similar with *pt_i_*. Let *dis*(*pt_i_*) be the set of causative genes of the phenotype *pt_i_*. We define the seed disease gene set with respect to *pt_i_* as 
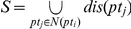
. For a seed disease gene *s∈* 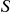, we assign to it a score 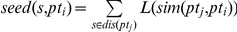 [24]. Given a phenotype *pt_i_* and the score for its seed gene set 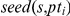, we can then score the protein complex *c_A_* as follows:
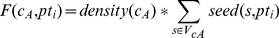
(9)
*F*(*c_A_*, *pt_i_*) denotes initial score of the protein complex *c_A_* with respect to *pt_i_*.

The density of a graph *G = (V_G_, E_G_)*, denoted as *density* (*G*), quantifies the richness of edges within *G* and it is defined as shown in equation (10) [25]:
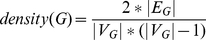
(10)Note that 0≤*density* (*G*)≤1. If *density* (*G*) = 1, then *G* is a complete graph, which means every pair of distinct vertices in *V_G_* is connected by an edge. As each protein complex can be viewed as a graph, we apply *density(C_A_)* to quantify the richness of protein interactions within *C_A_*. Those complexes with higher densities and their component proteins have multiple associations with query phenotypes and/or their phenotypic neighbors will get higher scores.

#### Step 2. Propagating the seeds' influence to the complexes in the whole network

We adopt the Random Network algorithm [26] to the protein complex network. First, the seed protein complexes are each assigned a score with respect to the query phenotype if they contain the genes in the seed disease gene set. We then score all the protein complexes in *COM* by propagation. We propose to do flow propagation for this. The prior disease influence flows of seed complex vertices are distributed and pumped to their neighbor complexes in the network. These super vertices will then continue to spread the influence flows received from previous iteration to their neighbors.

Formally, let *F_0_* be a vector of the initial probabilities of all the protein complexes in the protein complex network computed using equation (9). The probability vector at step *r*, *F_r_*, can be calculated by equation 11,
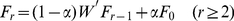
(11)where *F_1_* = *F_0_*.

*W'* is the column normalized form transpose of adjacency matrix *W* which is the transition matrix of the whole protein complex interaction network. We construct matrix *W* based on the three different links between protein complexes. Recall that our protein complex set *COM* consists of both multi-protein complexes (*C_M_*) and individual complexes (*C_I_*). The matrix *W* is thus defined as:
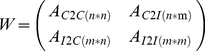
(12)where *A_C2C_*_ (n*n)_, *A_C2I_*_ (n*m)_, *A_I2C_*_ (m*n)_ and *A_I2I_*_ (m*m)_ are the adjacency sub-matrices. In particular, *A_C2C_*_ (n*n)_ represents the sub-network links between multiple-protein complexes (equation 5), *A_C2I_*_ (n*m)_ represents the sub-network links from multi-protein complexes to individual complexes, *A_I2C_*_ (m*n)_ represents the sub-network links from individual complexes to multi-protein complexes (equation 7), and *A_I2I_*_ (m*m)_ represents the sub-network links between individual protein complexes (equation 8) respectively, where *n = *|*C_M_*| and *m = |C_I_|* are the numbers of multi-protein complexes and individual complexes respectively.

Note that in Equation 11, the parameter 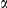
∈(0,1) provides a probabilistic weighting of spreading the prior information of the seed complex vertices to other protein complexes at every step. 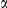 is set as 0.8 in our experiments. At the end of the iterations, the prior information held by every vertex in protein complex network will reach a steady state which is proven by paper [26]. This is determined by the probability difference between *F_r_* and *F_r-1_*_,_ represented as *Dif = *|*F_r_*−*F_r-1_*| (measured by *L1* norm). When *Dif* = |*F_r_*−*F_r-1_*|< = 10^−10^, as suggested in Li *et al.* [3], we consider that a steady stage has been reached and stop the iterative process. Note that the function *F* is smooth over the whole protein complex-complex network, and each vertex complex is assigned a value to represent its association with the disease phenotype of interest.

#### Step 3. Scoring disease gene based on associations of protein complexes to diseases

Once the vector *F_r_* reaches a steady state, we obtain the final scores of protein complexes with respect to query phenotype. Recall that the final objective of our algorithm is to prioritize candidate disease genes amongst the genes in the *G_PPI_*. The final step is therefore to prioritize candidate disease genes based on their associations with protein complexes. Given a candidate gene *g*, its association with query phenotype *pt_i_*, denoted by *S*(*g*, *pt_i_*), is computed as
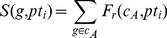
(13)where *C_A_* is the set of complexes containing the gene *g*, *F_r_(c_A_, pt_i_)* denotes probability of complex *C_A_* associated with phenotype *pt_i_* when *F_r_* reaches a steady state. Because mutations on the genes shared by multiple protein complexes may lead to multiple similar phenotypes, scores of these shared genes should be the accumulated score of protein complexes that contain them.

## Results

In this section, we firstly introduce the experimental settings and evaluation metrics. Then, we present the experimental results compared with state-of-the-art techniques.

### Experimental settings and evaluation metrics

Our objective is to uncover novel gene-phenotype relationships. In order to compare different techniques, we employ standard leave-one-out cross-validation in our experiments. Each known gene-phenotype association (*g*, *p*) is employed as one test case where the phenotype *p* is the query phenotype and the gene *g* is the test disease gene. In each round of cross-validation test, we will first intentionally remove the association (*g*, *p*) from our data. We then run our proposed algorithm to score the genes based on their associations with protein complexes with respect to the query phenotype *p*. If the test disease gene *g* is ranked as top 1, we will consider it as a successful prediction; otherwise it is a failed case. We use the number of overall successful predictions to evaluate the performance of different prediction methods. Depending on the genes involved in the ranking, we further categorize our evaluation metrics into the following two classes, namely, *whole genome* evaluation and *ab initio* evaluation [15]. *Whole genome* evaluation proposed by [15] basically ranks all the genes to scan for disease genes, e.g. we can consider all HPRD genes which do not link to the query phenotype (exactly same setting with RWRH [3]) and check how many known test disease genes are still ranked as top 1 in the cross-validation test. However, there are no causative genes for half of the OMIM phenotypes [4]. *Ab initio* prediction proposed by Wu [15] identifies disease genes without any known disease genes for those query phenotypes. For each phenotype entity, we remove the gene-phenotype associations from this phenotype *p* to all of its known causative genes and we can only use the other disease genes associated with *p*'s neighbor phenotypes as the seed disease gene set. If one of the known causative genes (assuming *p* is associated with multiple disease genes) related to the phenotype *p* is ranked top 1, we consider it a successful prediction.

Note in our experiments, the same experimental data and evaluation metrics have been consistently used to evaluate all the prediction techniques.

### Experimental Results

In this section, we first compare our algorithm with two state-of-the-art techniques, namely, CIPHER-DN (CIPHER with the topological distance feature of Direct Neighbors) [15] and RWRH [3]. Next, we test the sensitivities of the parameters in our proposal method. For discussion, we present two case studies of predicting disease genes for two representative diseases i.e., Breast cancer and Diabetes. Finally, with the computed scores for protein complexes, we want to validate if those the protein complexes with high scores are disease related.

#### Comparison with CIPHER-DN and RWRH

We compared the performance of our RWPCN algorithm with current computational techniques, namely, CIPHER-DN and RWRH, using two evaluation metrics presented above, namely, *whole genome* evaluation and *ab initio* evaluation. Table 1 shows the overall comparison results of different algorithms. In terms of *whole genome* evaluation (second column in Table 1), we observed that our proposed RWPCN was able to achieve the best result, successfully predicting 253 genes, which were 8 and 88 more genes predicted than RWRH and CIPHER-DN respectively. In terms of *ab initio* evaluation (third column in Table 1), we were able to predict 226 disease genes successfully, which were 25 and 69 more than the RWRH and CIPHER-DN respectively.

**Table 1:** Overall performance of RWRH, CIPHER-DN and RWPCN algorithm.

Algorithm	Whole genome evaluation	Ab initio evaluation
RWPCN	253	226
RWRH	245	201
CIPHER-DN	165	157

We compared RWPCN with RWRH and CIPHER-DN based on the measurement of whole genome evaluation and ab initio evaluation.

Note that in the original CIPHER-DN paper [15], the authors have adopted a less strict evaluation metric for *ab initio* evaluation than ours. As long as the target gene was ranked among the top *N* (*instead of the top 1*), it was regarded as a successful prediction where *N* (*N*> = 1) denotes the number of known disease genes for the query phenotype. Using this less stringent evaluation metric, our method predicted 240 genes successfully while CIPHER-DN could only predict 157 genes in the *ab initio* evaluation.

In the evaluations above, we have used the standard (but old) gene-phenotype association data which were also used in [3], [15] for comparison purpose. To further validate the predicted associations, we collected a new version of gene-phenotype association data extracted from OMIM using BIOMART recently [22]. It contains 1614 gene-phenotype associations, which includes 274 novel gene-phenotype associations where the disease genes were unknown in the previous version (other 1340 associations are shared by both versions). Table 2 shows that using the new gene-phenotype association data, RWPCN successfully ranked the 273 (a sensitivity of 0.169) genes as top 1 in terms of *whole genome* evaluation, and 247 (a sensitivity of 0.153) in terms of *ab initio* evaluation, indicating our method is certainly capable of detecting the novel knowledge which were absent in the older reference data.

**Table 2:** Overall performance of BIOMART06, 09 and 06+09 phenotype-gene data.

Phenotype-gene data	Whole genome evaluation	Ab initio evaluation
BIOMART06	253	226
BIOMART09	273	247
BIOMART06+09	285	253

We ran RWPCN on three kinds of phenotype-gene association data, extracted from BIOMART 06, BIOMART 09 and combination of two version data.

#### Effect of parameters 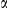 and k in RWPCN

Recall that we have two parameters 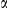 and *k* in our RWPCN algorithm. The flow parameter 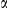 is used in our RWPCN algorithm to control the proportion of information that flows back into the seed nodes/protein complexes at each iteration of the algorithm. A larger 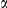 represents that information flows are likely to return to the seed nodes, therefore those protein complexes near to seed nodes are more likely to be ranked forward. On the contrary, a smaller 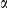 represents that information flows are likely to flow out of the seed nodes, therefore those protein complexes near to seed nodes are more likely to be ranked backward. The second phenotype parameter *k* decides the number of related phenotypes with regard to the query phenotype. An unnecessarily large *k* will include many phenotypes which are not relevant while a smaller *k* will include lesser number of related phenotypes and may miss out some important relevant phenotypes as a result.

**Figure 2:**
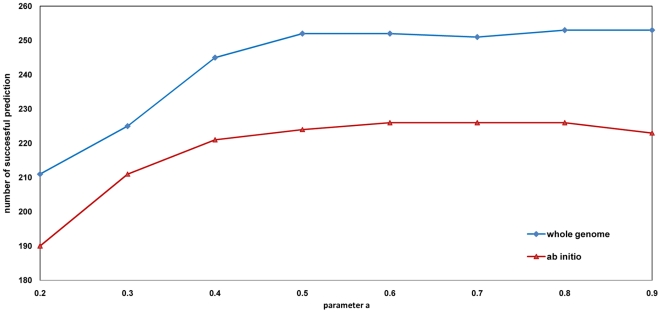
Effect of value 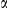 based on whole genome and ab initio evaluation. Figure 2 investigated how the flow parameter affects the performance of the RWPCN algorithm. With increasing value of 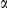, we can obtain increased numbers of successful predictions for both *whole genome* evaluation and *ab initio* evaluation.

We first investigated how the flow parameter 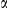 affects the performance of the algorithm. We ran our algorithm with values of 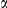 ranging from 0.2 to 0.9 in steps of 0.1, while keeping the phenotype *k* fixed as 10 using leave-one-out cross-validation. The performance of the algorithms is measured using *whole genome* evaluation and *ab initio* evaluation mentioned above, as shown in Figure 2.

With increasing value of 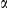, we were able to obtain increased numbers of successful predictions for both *whole genome* evaluation and *ab initio* evaluation. This is expected since the seed nodes in protein complex network are more likely to hold the information flows, thus few flows will be distributed to the distant neighbors in the network. Biologically, this is reasonable since the protein complexes (and the corresponding proteins in the complexes) that directly interact with the disease complexes/proteins are more likely to be disease/phenotype related. We observe that the performance of RWPCN with 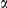
> = 0.4 are better than the existing CIPHER-DN and RWRH algorithms. In fact, we found that the optimal values of 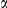 can be found within a large range of 0.5< = 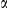
< = 0.9. As such, selecting a suitable value for 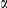 for good performance is not a problem.

**Figure 3:**
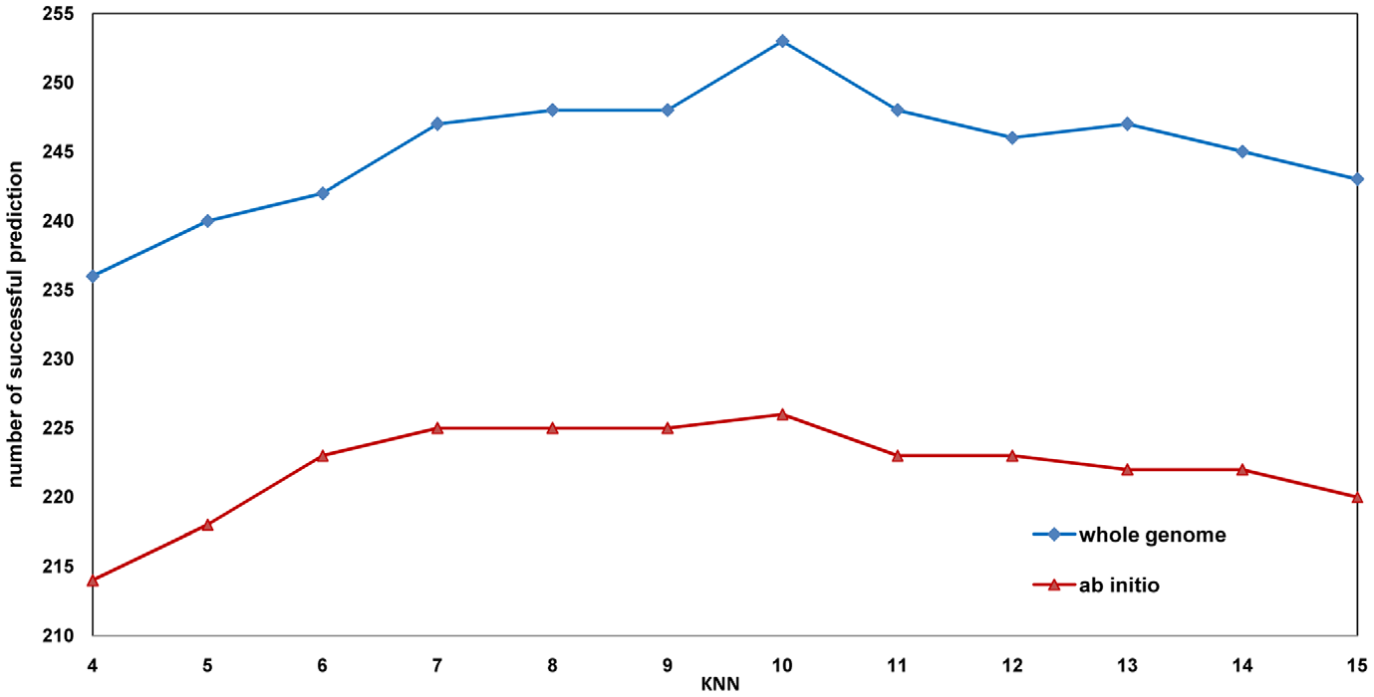
KNN phenotype network on whole genome and ab initio evaluation. This figure studies the effect of the parameter *k* that decides the number of related phenotypes. Figure 3 shows the performance of RWPCN algorithm improved with increasing value of *k* from 4 to 10 (incorporating more related phenotypes) but it performs worse than when *k*>10 (including low noisy phenotypes). Overall, the performance of RWPCN algorithm with *k* in the wide range [7], [12] was consistently better than that of RWRH and CIPHER-DN.

To study the effect of the parameter *k* that decides the number of related phenotypes, we ran RWPCN with *k* from 4 to 15 with 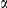 = 0.8, based on *whole genome* and *ab initio* evaluations. Results are shown in Figure 3. The performance of RWPCN algorithm improved with increasing value of *k* from 4 to 10, indicating that incorporating more related phenotypes is helpful for prioritizing target disease genes. However, if we further include more phenotypes (e.g. when *k*>10) with low phenotypic similarities, noisy and un-meaningful phenotypes will be included [23] and eventually affects the performance of disease gene prediction. For example, the results in Figures 3 showed that the performance with *k* in the range of [11], [15] has worsened. Nevertheless, the performance of RWPCN algorithm with *k* in the wide range [7], [12] was consistently better than that of RWRH and CIPHER-DN, suggesting that RWPCN is insensitive to the specific values of *k*.

#### Inferring novel causal genes for breast cancer and diabetes

We also applied our method for uncovering novel candidate genes on specific complex genetic diseases. We have chosen Breast Cancer (MIM: 114480) and Diabetes Mellitus type 2 (MIM: 125853) for our case studies here.

We ran our RWPCN algorithm (with *k* = 10 and 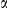 = 0.8) for both Breast Cancer and Diabetes Mellitus type 2. Note that we no longer removed any gene-phenotype associations since our objective was to predict *novel* disease genes instead of evaluating the performance using cross-validation. We ranked the resulting candidate genes over the whole genome and selected the top 20 ranked genes associating with target phenotypes (Breast Cancer and Diabetes Mellitus type 2).

The experimental results are listed in Table 3 and 4 for Breast Cancer and Diabetes Mellitus type 2 respectively.

**Table 3:** Breast cancer genes prediction.

*Rank*	*Score*	*HGNC Gene symbol*	*Mark*
1	3.61665	BRCA1	*
2	2.64458	RBBP8	√
3	1.04115	HDAC1	√
4	1.02108	HDAC2	√
5	1.00632	CTBP1	∼
6	0.983392	LMO4	√
7	0.814445	RAD51	*
8	0.812762	BRCA2	*
9	0.807072	NBN	*
10	0.806886	BRIP1	*
11	0.801356	PIK3CA	*
12	0.671104	ZNF350	√
13	0.142519	SMAD3	∼
14	0.141945	ELAC2	√
15	0.141729	RNASEL	√
16	0.140748	PTEN	√
17	0.0947266	TP53	∼
18	0.0849672	SMAD4	∼
19	0.0831955	EP300	∼
20	0.0721527	CREBBP	∼

Genes marked with * are known disease genes associated with Breast Cancer (MIM: 114480), genes marked with √ are the genes associated with Breast Cancer (MIM: 114480) either extracted from literature or from GENECARDS database, genes marked with ∼ are un-related to disease.

**Table 4:** Diabetes genes prediction.

*rank*	*Score*	*HGNC Gene symbol*	*Mark*
1	1.34591	PIK3R1	√
2	1.33691	IRS1	*
3	1.33691	INSR	*
4	1.33691	KHDRBS1	∼
5	0.821847	NEUROD1	*
6	0.812877	IPF1	*
7	0.810154	SLC2A4	*
8	0.802705	MAPK8IP1	*
9	0.802453	TCF2	*
10	0.802404	PPP1R3A	*
11	0.354724	TCF1	∼
12	0.194629	CREBBP	∼
13	0.15557	EP300	√
14	0.102423	PCAF	∼
15	0.0807789	PLN	∼
16	0.0806853	RPS6KA1	∼
17	0.0652625	CUL3	∼
18	0.0652625	SPOP	∼
19	0.0595811	POLR2A	∼
20	0.0471911	ABCC8	√

Similarly, Genes marked with * are known disease genes associated with Diabetes Mellitus, type 2 (MIM: 125853), genes marked with √ are the genes associated with Diabetes Mellitus, type 2 (MIM: 125853), extracted from literature or from GENECARDS database, genes marked with ∼ are un-related with disease.

Table 3 showed 6 highly ranked genes that are also known to associate with the Breast Cancer. However, we are more interested in investigating whether our predicted *novel* susceptible genes are also associated with disease phenotypes. We searched for additional gene-phenotype associations from GENECARDS database [27] and also performed literature search from PubMed on the other susceptible genes predicted by our algorithm to be associated to disease phenotype of Breast Cancer (MIM: 114480). We found 8 additional genes, namely RBBP8, HDAC1, HDAC2, LMO4, ZNF350, ELAC2, RNASEL and PTEN that are also reported to be related to Breast Cancer. For CtIP (also known as retinoblastoma binding protein 8, RBBP8, ranked at 2), the expression of this gene had been shown to be a novel mechanism for tamoxifen resistance development in breast cancer [28]. HDAC1 and HDAC2 (ranked at 3 and 4), among class I HDACs, were reported to regulate the changes in histone acetylation and were associated with HDAC inhibitors that were expected to reverse hypoacetylation levels observed even at the early stages of breast cancer progression [29]. LMO4 (ranked at 6) was a novel cell cycle regulator with a key role in mediator of ErbB2/HER2/HER2/Neu-induced breast cancer cell cycle progression [30]. Genetic variants and haplotype analyses of the ZNF350 (ranked at 12) gene suggested that it is associated with high-risk non BRCA1/2 French Canadian breast and ovarian cancer families [31]. Germline mutation in RNASEL (ranked at 15) predicted increased risk of breast cancer [32]. Finally, Tsou HC et al. [33] reported three novel MMAC1/PTEN (ranked at 16) mutations in CS (Cowden syndrome) were associated with breast cancer. All these showed that our prediction method could discover novel disease genes for breast cancer beyond the original disease gene set.

Table 4 showed our prediction results for Diabetes Mellitus type 2. Out of the top 20 predicted disease genes, 8 genes were known to associate with the phenotype. We found three additional genes PIK3R1, EP300 and ABCC8 also related to the disease phenotypes. PIK3R1 (ranked at 1) had been tested for their influence on insulin action, showing significant associations with diabetes [34]. EP300 (ranked at 13, aliases p300), as a transcriptional coactivator, could cause diabetes via regulating fibronectin expression via PARP and NF-kappaB activation [35]. For ABCC8, a rare mutation in ABCC8/SUR1 (ranked at 20) had been reported to have an effect on K(ATP) channel activity and beta-cell glucose sensing, leading to diabetes in adulthood [36].

From Tables 2 and 3, we found our predicted disease genes indeed mapped significantly with disease genes that were either curated in existing databases or reported in the literature. This suggests that the other unmatched ones could be potentially real disease genes that are worth being further validated by clinicians and biologists.

#### Detecting disease-related protein complexes

Recall that we have assigned scores to the protein complexes to indicate the degree of association of the protein complexes to the query disease phenotypes. The higher the scores protein complexes were assigned, the higher probability protein complexes were associated with corresponding phenotypes. Based on the scores, we ranked the protein complexes and studied the two top-ranked complexes here: Sarcoglycan-sarcospan complex (*SG-SPN*) and Pex26-Pex6-Pex1 complex. For evaluation, a set of 248 disease protein complexes from Lage *et al.* [18] was used as our benchmark.

**Figure 4:**
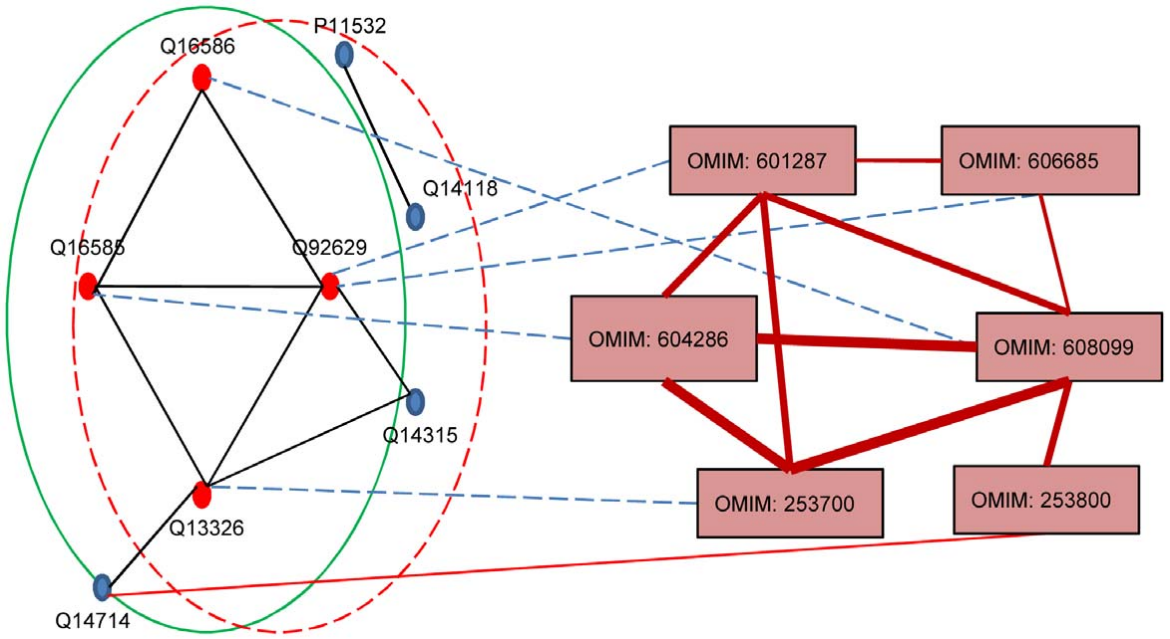
SG-SPN overlaps with the disease complex No. 230. Figure 4 showed that the *SG-SPN* complex (surrounded by green line) contained five human proteins. We found that this SG-SPN complex had a large overlap (shared four proteins) with the disease complex No. 230 (surrounded by red dash line) in our benchmark set. We also found that all shared 4 proteins were known disease genes linked to phenotypes (Blue dash links), which had high phenotypic similarities among them. Gene Q14714 (SSPN) in SG-SPN complex was associated with phenotype Fukuyama Congenital Muscular Dystrophy (FCMD) (MIM: 253800) [37] which was closely related to phenotype (MIM: 608099) in our phenotype network.

Figure 4 showed that the *SG-SPN* complex (surrounded by green line) contained five human proteins: Q16586, Q16585, Q92629, Q13326, Q14714 and it was ranked at top 1 protein complex in predicting phenotype (MIM: 608099)-gene (SGCA) association by our RWPCN algorithm. We found that this SG-SPN complex had a large overlap (shared four proteins) with the disease complex No. 230 (surrounded by red dash line) in our benchmark set. We also found that all shared 4 proteins were known disease genes linked to phenotypes (Blue dash links), which had high phenotypic similarities among them. Noted that gene Q14714 (SSPN) in SG-SPN complex was associated with phenotype Fukuyama Congenital Muscular Dystrophy (FCMD) (MIM: 253800) [37] which was closely related to phenotype (MIM: 608099) in our phenotype network, indicating that *SG-SPN* complex could indeed be a valid disease complex.

**Figure 5:**
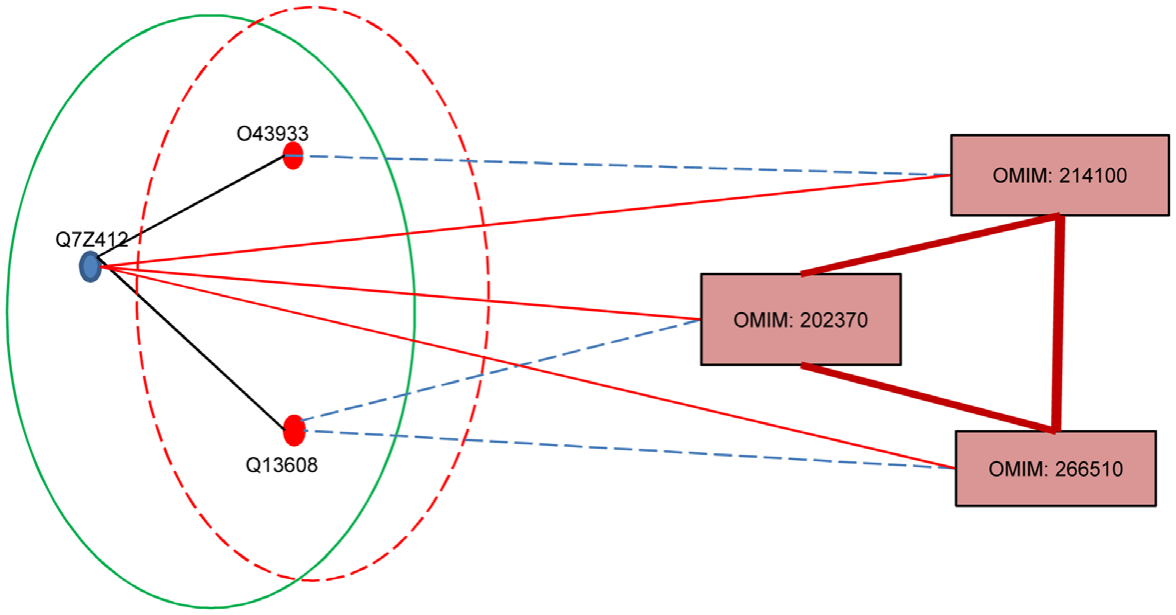
Pex26-Pex6-Pex1 overlaps with the disease complex No. 335. This figure showed the Pex26-Pex6-Pex1 complex (surrounded by green line) which covered a benchmark disease complex (surrounded by red dash line) that consisted of proteins O43933 (PEX 1) and Q13608 (PEX 6). The Pex26-Pex6-Pex1 complex was involved in peroxisome biogenesis disorders (PBDs), which included the Zellweger syndrome spectrum (*PBD-ZSD*) and rhizomelic chondrodysplasia punctata type 1 (RCDP1). *PBD-ZSD* represented a continuum of disorders including infantile Refsum disease (MIM: 266510), neonatal adrenoleukodystrophy (MIM: 202370), and Zellweger syndrome (MIM: 214100).

Similarly, Figure 5 showed the Pex26-Pex6-Pex1 complex (surrounded by green line) which covered a benchmark disease complex (surrounded by red dash line) that consisted of proteins O43933 (PEX 1) and Q13608 (PEX 6). This complex was ranked at top 1 in inferring phenotype (MIM: 202370)-gene (PEX26) association. The Pex26-Pex6-Pex1 complex was involved in peroxisome biogenesis disorders (PBDs), which included the Zellweger syndrome spectrum (*PBD-ZSD*) and rhizomelic chondrodysplasia punctata type 1 (RCDP1). *PBD-ZSD* represented a continuum of disorders including infantile Refsum disease (MIM: 266510), neonatal adrenoleukodystrophy (MIM: 202370), and Zellweger syndrome (MIM: 214100). Noted that the Q7Z412 (PEX 26) protein in the our predicted disease complex was also a known disease gene associated with all the three phenotypes, suggesting that the mutations of proteins in the same CORUM protein complexes were likely to induce the same or similar phenotypes. It also showed that our highly ranked protein complexes were indeed disease related.

## Discussion

While great progress has been made in genomics and proteomics, discovering the associations between genes and phenotypes have remained challenging. In this paper, we constructed a novel human protein complex network by integrating HPRD protein interaction network [20] and CORUM protein complexes [19]. We showed that a genome-wide disease gene prioritization for multi-factorial diseases can be obtained using such a human protein complex network. Using our method, the top ranked candidate disease genes that are found to be closely associating with protein complex can potentially be used to guide the prediction of disease-related protein complexes.

We have verified the ability of our RWPCN algorithm to disclose gene-phenotype associations through extensive experiments. We first exploit known gene-phenotype associations to initialize the higher-level complex phenotype associations based on the modular nature of complex diseases. Then, we prioritize candidate disease genes for disease phenotypes using the network propagation technique on the complex interaction network. Our RWPCN algorithm was shown to outperform the existing methods RWRH [3] and CIPHER [15] which only use gene/protein level associations. This suggests that our protein complex network can indeed capture the underlying modularity in the biological interaction networks better than simple protein interaction networks, as it is a more effective basis for interrogating the human phenome-interactome network for gene-phenotype associations through our RWPCN algorithm.

It should be acknowledged that the proposed RWPCN algorithm can be improved further. As RWPCN relies on the human protein complex interaction network, the coverage of the protein complex data can affect the performance of prediction. Since the current protein complex data is by no means complete, predicted human protein complexes with high quality could be taken into consideration. Combining the predicted and experimental validated complex data into the prioritization process (e.g. using the method reviewed in [38]–[40]), could increase the power of prediction as long as we also ensure the quality of the complex data. RWPCN also depends on the quality (i.e. reliability) of the PPI data in the current model, and it may be more suited to certain kind of diseases than others. It is well-known that PPI data generated with high-throughput methods can be of inferior quality. One possible improvement is to weight protein-protein interactions using diverse biological evidences (e.g. protein sequences, domain, motif, topological properties of PPI network [41], [42], protein localization, molecular function, biological process and gene expression profiles [43], [44], metabolic reactions [45], etc) to improve the reliability of the PPI data that we use for disease gene prioritization. We are currently exploring these and other approaches to further improve our RWPCN algorithm for discovering gene-phenotype associations.
